# Agomelatine Efficacy in the Night Eating Syndrome

**DOI:** 10.1155/2013/867650

**Published:** 2013-05-16

**Authors:** Walter Milano, Michele De Rosa, Luca Milano, Anna Capasso

**Affiliations:** ^1^Mental Health Unit, District 24, ASL Napoli 1 Centro, Molosiglio, Via Acton, 80145 Napoli, Italy; ^2^Department of Pharmacy, University of Salerno, Via Ponte don Melillo, Fisciano, 84084 Salerno, Italy

## Abstract

Night eating syndrome (NES) is a nosographic entity included among the forms not otherwise specified (EDNOS) in eating disorders (ED) of the DSM IV. It is characterized by a reduced food intake during the day, evening hyperphagia, and nocturnal awakenings associated with conscious episodes of compulsive ingestion of food. Frequently, NES patients show significant psychopathology comorbidity with affective disorders. This paper describes a case report of an NES patient treated with agomelatine, an antidepressant analogue of melatonin, which acts by improving not only the mood but also by regulating sleep cycles and appetite. After three months of observation, the use of Agomelatine not only improved the mood of our NES patient (assessed in the HAM-D scores) but it was also able to reduce the night eating questionnaire, by both reducing the number of nocturnal awakenings with food intake, the time of snoring, the minutes of movement during night sleep (assessed at polysomnography), and the weight (−5.5 kg) and optimizing blood glucose and lipid profile. In our clinical case report, agomelatine was able both to reduce the NES symptoms and to significantly improve the mood of our NES patient without adverse side effects during the duration of treatment. Therefore, our case report supports the rationale for further studies on the use of Agomelatine in the NES treatment.

## 1. Introduction

The night eating syndrome (NES) is a disorder at the time, between otherwise specified forms of eating disorders. NES is characterized by a reduced feeding during the day, evening hyperphagia accompanied by frequent nocturnal awakenings associated with conscious episodes of compulsive ingestion of food [1, 2]. NES is characterized by an abnormal circadian rhythms of food and other neuroendocrine factors. Frequently it is associated with obesity and depression mood [1, 2].

The night eating syndrome (NES) is counted in the DSM IV [3] not otherwise specified in the forms of eating disorders (ED) although, along with binge eating disorder (BED), NES is now considered a disorder worthy of entity separate from its clinical nosographic dealing with these specific diseases food patterns, including its close links with obesity [4].

Allison et al. [5] proposed new criteria for diagnosis of the NES. This research has established two core criteria: (1) the consumption of at least 25% of daily caloric intake after the evening meal and/or (2) evening awakenings with ingestions at least twice per week. Five descriptors have been added to the core criteria, three of which are required for the diagnosis of NES. Additionally, people must be aware of their nocturnal ingestions; they must experience distress or impairment in functioning, and they must have experienced the signs and symptoms for the past 3 months [5]. These criteria help standardize the definition of NES. Additional aspects of the nosology of NES yet to be fully elaborated include its relationship to other eating and sleep disorders. Assessment and analytic tools are needed to assess these new criteria more accurately [5].

In this way, the NES can be clearly distinguished from other related diseases such as binge eating disorder or sleep-related eating disorder. The authors conclude that a clearer connotation nosographic allows a better definition for the prevalence, its association with obesity, the assessment of the frequent comorbidity, and a more effective determination of the underlying biological implications [5]. Therefore, NES appears to be a combination of an eating disorder, a sleeping disorder, and a mood disorder [4–6].

Altering the timing of food intake, typical of NES, is related to abnormal neuroendocrine patterns. The blood levels of cortisol, albeit measured on a limited number of studies, are on average higher and lower circadian fluctuations and appear to increase the production of TSH [7, 8], similar to stress-related disorders [9]. 

Several studies also show that people with the NES have lowered levels of melatonin which is the naturally occurring hormone that regulates the body's circadian rhythms that control the biochemical, the physiological, and the behavioral 24-hour cycles such as sleep and many others [7]. Therefore, it is believed that the decreased melatonin is a big contributor to disturbances of sleep and the onset of the night eating syndrome. Additional factors that contribute to the NES and its nocturnal ingestion are leptin [7–9] (the hormone that is believed to suppress appetite and speed up metabolism), certain medications, and highly restrictive and prolonged dieting among obese individuals. Even the regulation of ghrelin [9], an endogenous ligand receptor growth hormone (GH), which affects not only food but also control induction of sleep, is altered in the NES [9]. 

Emotional factors such as depression, anxiety, stress, boredom, low self-esteem, and skewed body image play a significant role in the NES, and they are the catalysts that lead to night binging on comfort foods that have high caloric values from their carbohydrates and fat contents [10]. Often in patients with NES are found, and in patients with other eating disorders, significant comorbid psychopathology such as depression. Patients with NES often have higher scores on Beck Depression Inventory Scale and the Zung Depression Scale compared with controls [10] with a chance of lifetime incidence of major depression of 55% DM [11]. But often in patients with NES there is a decline in mood in the evening and at night, in the opposite way to experience clinical depression typical [12]. There are also frequent, although symptoms related to a condition of anxiety and worthlessness [13, 14]. 

The NES is treatable, but it is not easy. In this respect, treatment of NES, because of the complexity of diagnosis, has to be done on an individual basis, combining mental health therapy, education on diet and nutrition, possibly medication to reduce stress, time spent in a sleep lab for observation, and a great deal of support. This particular disorder is showing signs of responding favorably to the antidepressants such as selective serotonin reuptake inhibitors (SSRIs). These have been found useful due to their effect on the serotonin levels in the brain. Serotonin promotes calm, helps counteract cravings, and is involved in the production of melatonin which aids sleep. 

However, the current trials on pharmacological treatments of NES are still at a preliminary stage. Some literature data suggest that three types of drugs seem likely to be effective in reducing episodes of NES:

1. dopaminergic drugs such as pramipexole [15],
2. anticonvulsants such as topiramate [16],
3. selective serotonin reuptake inhibitors (SSRIs) [17–19].

According to recent evidence, it would seem useful to associate with drug therapy a cognitive behavior therapy (CBT) [20, 21]. 

Given the strong relationship between NES, cycles sleep, and food alteration, as well as the frequent presence of depressed mood, associated with lower circulating levels of melatonin in the evening and at night, the treatment of NES with agomelatine, a melatonin agonist, may be considered.

**Figure 1:**
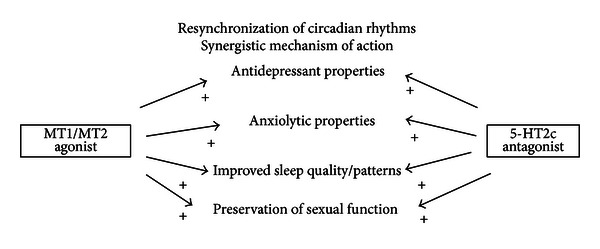
Mechanism of action of Agomelatine.

Agomelatine is a selective agonist of melatonin receptors MT1 and MT2, present in various areas of the brain, including the hypothalamic suprachiasmatic nucleus, the substantia nigra, hippocampus, and nucleus accumbens, but it is also an antagonist of serotonin 5-HT2C receptors [22, 23] (Figure 1). Agomelatine has also a significant anxiolytic and antidepressant action because of the synergy between the agonist effect on receptors for melatonin and serotonin antagonist. The activation of the MT1 and MT2 receptors in the suprachiasmatic nucleus can modulate the functions and therefore most importantly normalize circadian rhythms, including sleep-wake cycle [23], while the selective blockade of receptors 5HT2c, located at the level of termination noradrenergic and dopaminergic agents, can enhance the function of these two neurotransmitters in the frontal cortex, hippocampus, and other brain areas which play a crucial role in the modulation of cognitive, affective, and emotional [24]. In addition, some evidence indicates that agomelatine is an effective inducer of the process of neurogenesis and, more generally, neuronal plasticity through an increase in brain derived neurotrophic factor (BDNF) [24–27]. Therefore, in the present paper, we report the effect of agomelatine in one of our NES patient.

## 2. Participant and Procedures

At the Mental Health Unit of the District 24 ASL Napoli 1 Centre, we have followed a female patient (MR. R.; 39-year old) affected by the NES by 5 years. The NES patient reported frequent nocturnal awakenings associated with consumption of food, especially sweets and snacks, with a marked decrease in appetite during the day and a hearty evening meal. Also, she showed a marked increase in weight over the past 5 years, about 12 kg, (BMI from 23.5 to 28). The mood was significantly depressed. For about two years, she practiced antidepressant therapy, first with Venlafaxine 150 mg/day and then with Sertraline 100 mg/day. During this period of therapy, the mood had improved considerably but the awakenings and episodes of nocturnal feeding remained almost unchanged. In January 2011, the patient discontinued treatment with Sertraline and the following March went to a treatment with Agomelatine, first with 25 mg/day and then after 10 days with 50 mg/day.

### 2.1. Assessment Measures

The study involved the administration of two scales, before and after observation: the night eating questionnaire (NEQ) [28] and hamilton depression rating scale-HDRS or HAM-D [29]. Both measures are self-administered and take about an hour to administer both. The NEQ consists of 15 items and participants who reported on the NEQ consuming ≥25% of their caloric intake after their evening meal or the presence of nocturnal ingestions (waking after sleep onset to eat) were asked follow-up questions to confirm the extent of evening hyperphagia, frequency of nocturnal ingestions, and the context of their night eating behavior. The NES interview included an outline of the general pattern of food intake for a typical 24-hour period and a specific recall of all food consumed after the evening meal 2 nights before the interview, provided it was representative of a typical evening's food intake. 

Hamilton depression rating scale (HDRS), or abbreviated to HAM-D, is a multiple choice questionnaire that clinicians may use to rate the severity of a patient's major depression. The scale examines 21 different areas that are critical to the evaluation of the depressive state of the subject. The areas are depressed mood, guilt, suicidal ideation, initial insomnia, middle insomnia, insomnia, prolonged work and interests, slowing of thought and words, agitation, anxiety, psychic source, somatic anxiety, gastrointestinal somatic symptoms, general somatic symptoms, genital symptoms, hypochondriasis, introspection, weight loss, diurnal variation of symptoms, depersonalization, paranoid symptoms, and obsessive symptoms. 

Each of the 21 areas represents an individual scale items.

### 2.2. Polysomnographic Recordings

It is an instrumental test that records during sleep the presence of snoring, heart rate and electrocardiogram, body position of the subject, the saturation of blood, the respiratory movement, and airflow. It is a test that is performed at the patient's home. The execution of polysomnography involves applying three electrodes to the chest, a finger pulse oximeter, and a breath meter outside the nose during sleep, a small computer (Embletta Sonografica) to which all sensors are connected with the main parameters of respiration and heart activity.

## 3. Results

Before starting therapy with agomelatine, our patient had a score to the night eating questionnaire (NEQ) [26] of 38 and 20 of the hamilton rating scale for depression (HAM-D) and about four awakenings with food intake per night on average with power. Polysomnographic examination episodes of apnea and hypopnea were normal: 11 per night, with a ratio apnea-hypopnea index (AHI) of 1.3/h (nv < 5), snoring time of 28 minutes, equal to 5, 4% of total sleep time (510 minutes), movement time of 25 minutes, 4.9% of total sleep time (510 minutes) (Table 1).

The weight was 70 kg (BMI 28 Kg/m^2^) and fasting blood levels were 118 mg/dL in the morning for blood sugar of 254 mg/dL for cholesterol of 186 mg/dL and 45 mg/dL for triglycerides to HDL cholesterol.

In June 2011, after three months of treatment, the patient reported a marked reduction in the number of nocturnal awakenings with food intake, with an average of less than two awakenings with food intake per night. The NEQ scores and the HAM-D were significantly reduced, respectively, to 25 and 9 (Table 1).

At polysomnography, the number of apnea and hypopnea episodes index remained normal, 12 per night with AHI of 1.7/h, the time snoring was reduced to 21 minutes, 4.4% of total sleep time (470 minute), and movement time was shortened to 15.2 minutes, 3% of total sleep time (470 minutes). The weight was reduced to 64.5 kg (BMI 26) and the values were 93 for blood glucose, cholesterol to 225, 132 and 48 for triglycerides to HDL cholesterol (Table 1).

## 4. Conclusions

Our study showed that agomelatine was able both to reduce the NES symptoms and to significantly improves mood of our NES patient without adverse side effects for the duration of treatment; therefore, it could be very useful in the NES treatment.

NES is often accompanied by changes not only of the circadian rhythms of sleep and food, with frequent awakenings associated with compulsive ingestion of food in non-REM stages of sleep [2], but also of alterations of cortisol secretion and other hormones [2]. Agomelatine is able to regulate the stressor-induced overactivity of the pituitary-adrenal axis (HPA), by improving the sleep slow wave (SW), without reducing REM sleep and by helping the synchronization of circadian rhythms [22, 23].

Also, thanks to the synergic action on receptors MT1 and MT2 agonist and 5-HT2C antagonistic properties (Figure 1), agomelatine is able to significantly improve mood and to reduce anxiety component, both frequently present in the NES comorbidities. In our case report, the use of agomelatine has helped our NES patient to reduce significantly the number of nocturnal awakenings with food intake (over 50%) and the number of minutes of movement during the night (from 4.9% to 3% per night), to improve the control of food intake during awakenings, as evidenced by improved scores on the NEQ (from 38 to 20). The reduction of food-intake led to a net loss of weight (−5.5 kg) and also to normalize some important blood values as blood glucose, total cholesterol, and triglycerides.

Furthermore, agomelatine improved both the mood of our NES patient, as evidenced by the reduction of the NAM-D score (from 20 to 9), and sleep quality, with less feeling of sleepiness and daytime fatigue. During the entire period of observation (3 months), there were no adverse side effects associated with the use of the drug. 

In conclusion, our case study supports the rationale for further studies on the use of agomelatine in the treatment of NES.

## Figures and Tables

**Table 1:** The effect of Agomelatine on a NES patient.

Age: 39 years old Sex: female	Before Agomelatine treatment	After Agomelatine treatment (3 months)
HAM-D score	20	9
NEQ score	38	25
Number of awakenings with food intake average per night	4	2
Weight Kg	70	64.5
BMI Kg/m^2^	28	26
Snoring time at polysomnography	28 minutes (5.4% of total sleep)	21 minutes (4.4% of total sleep)
Time moves at polysomnography	25 minutes (4.9% total sleep)	15.2 minutes (3% total sleep)
Glucose mg/dL	118	93
Cholesterol mg/dL	254	225
HDL cholesterol mg/dL	45	48
Triglycerides mg/dL	186	132
